# Traumatic injuries in drowning 

**Published:** 2022-01

**Authors:** Zahra Mohtasham-Amiri

**Affiliations:** ^*a*^ Guilan Road Trauma Research Center, Guilan University of Medical Sciences, Rasht, Iran.

## Abstract

Drowning is one of the most serious and neglected public health problems. It causes the loss of more than 200,000 lives annually. More than 90% of this mortality rate occurs in low- and middle-income countries. In 2019, injuries accounted for almost 8% of total global mortality. Drowning is the 3rd leading cause of unintentional injury deaths worldwide and children are the main at-risk group. Data on traumatic injuries in drowning is scarce in most developing countries due to lack of appropriate mortality reporting system. Moreover, most of the studies are limited to only a few countries. For every child who dies in a drowning accident, 5 others will receive hospital care. Out of these near-drowning cases, 15 percent will die, and 20 percent will be left with severe neurological injuries. Based on the current limited studies, the prevalence of drowning injuries varies from 0.5% to 11% because of the differences in the study populations and the levels of hospitals. All injuries occurred in a swimming pool. The most prevalent site of injuries due to drowning was cervical spine, injuries to the C5 through C6 levels were the most common. Anoxic brain injury secondary to submersion was the most cause of death in hospital. Also, studies mention isolated case reports of retinal hemorrhages. The physical injuries described included bruising and fractures. Old age and a history of diving were the most risk factors for getting injuries. It has been recommended that physicians and emergency health workers who care for drowning/near-drowning patients be aware of possible trauma, especially cervical spine injuries. Those patients with a history of a traumatic mechanism such as diving should undergo evaluation by specialized trauma teams.

**Keywords:**

Keywords: Injuries, Drowning, Near-drowning

